# Microglia in ALS: Insights into Mechanisms and Therapeutic Potential

**DOI:** 10.3390/cells14060421

**Published:** 2025-03-12

**Authors:** Silvano Bond, Smita Saxena, Julieth A. Sierra-Delgado

**Affiliations:** 1 Department of Physical Medicine and Rehabilitation, University of Missouri School of Medicine, Columbia, MO 65211, USA; sbnf8@missouri.edu; 2 NextGen Precision Health, University of Missouri, Columbia, MO 65211, USA

**Keywords:** ALS, microglia, neuroinflammation

## Abstract

Amyotrophic lateral sclerosis (ALS) is a progressive neurodegenerative disease characterized by the loss of motor neurons, leading to escalating muscle weakness, atrophy, and eventually paralysis. While neurons are the most visibly affected, emerging data highlight microglia—the brain’s resident immune cells—as key contributors to disease onset and progression. Rather than existing in a simple beneficial or harmful duality, microglia can adopt multiple functional states shaped by internal and external factors, including those in ALS. Collectively, these disease-specific forms are called disease-associated microglia (DAM). Research using rodent models, patient-derived cells, and human postmortem tissue shows that microglia can transition into DAM phenotypes, driving inflammation and neuronal injury. However, these cells can also fulfill protective roles under certain conditions, revealing their adaptable nature. This review explores recent discoveries regarding the multifaceted behavior of microglia in ALS, highlights important findings that link these immune cells to motor neuron deterioration, and discusses emerging therapies—some already used in clinical trials—that aim to recalibrate microglial functions and potentially slow disease progression.

## 1. Introduction

Amyotrophic lateral sclerosis (ALS), also known as “Lou Gehrig’s disease”, is a progressive and fatal neurodegenerative disorder that affects motor neurons in the brain and spinal cord, leading to muscle weakness, atrophy, and eventually paralysis [1]. Despite being the most common form of adult-onset motor neuron degeneration, ALS is considered a rare disease, with an incidence of approximately 4 per 100,000 people in the United States [2,3]. However, higher prevalence rates are observed in certain regions, with some areas reporting up to 7 per 100,000 [2]. The disease usually manifests in individuals between 40 and 65 years of age [1]. ALS is typically characterized by a focal onset which arises as unilateral distal muscle weakness and atrophy in upper or lower limb muscles (spinal ALS) or in bulbar muscles (bulbar ALS) that progressively spreads to the degeneration of all motor neurons, including those responsible for regulating functions dependent on the autonomic nervous system, such as respiration [4]. Among these subtypes, bulbar onset primarily affects speech and swallowing, while spinal onset predominantly impairs limb function. Progression states in ALS vary widely among patients and are influenced by factors such as disease subtype, genetic predisposition, and environmental exposure [4]. Some individuals experience a rapid decline, with motor neuron degeneration quickly leading to loss of function in multiple regions, while others exhibit a slower disease course with more gradual symptom onset and progression [1]. This variability can be observed in both bulbar and spinal ALS, where differences in the speed and pattern of motor neuron involvement significantly impact clinical outcomes and quality of life. Additionally, progression states often correlate with the burden of autonomic dysfunction, respiratory decline, and nutritional challenges, further complicating disease management. Some individuals experience a rapid decline, with motor neuron degeneration quickly leading to loss of function in multiple regions, while others exhibit a slower disease course with more gradual symptom onset and progression [1,2,3].

The evolving nature of ALS makes it fatal in 100% of cases over a period usually ranging between 2 and 5 years after diagnosis [1,2,3]. While ALS ultimately leads to the same outcome for all affected patients, it is becoming increasingly clear that the disease can be triggered by diverse factors and presents with a wide range of clinical symptoms. Currently, approximately 90% of ALS cases are sporadic (sALS), with risk factors including smoking, alcohol consumption, exposure to air pollutants, chemical agents, pesticides, certain types of trauma, and genetic mutations [4]. The remaining 10% are familial (fALS), although both forms are phenotypically indistinguishable [3].

Over 25 key genes have been implicated in ALS pathophysiology, including Superoxide Dismutase 1 (*SOD1*) [5,6], *C9ORF72* [7], and *p65 NF-κB* [7]. Mutations in these genes can lead to excitotoxicity [8,9,10], oxidative stress, neuroinflammation, and other pathogenic processes. Additionally, specific mutations in the *TDP-43* [11] and *FUS* [12] genes can cause the mislocalization and toxic aggregation of their protein products, which are observed in the motor neurons of nearly all ALS patients. These genetic factors contribute significantly to both sporadic and familial forms of ALS, underscoring the complexity of its pathogenesis [13].

Moreover, these mutations lead to RNA metabolism disruption and impairments in axonal transport, leading to significant dysfunction in highly susceptible motor neurons. While the involvement of genes and environmental factors in motor neuron impairment and death is well established, the roles of other cell types, such as microglia, astrocytes, and oligodendrocytes are becoming increasingly evident. Microglia, as part of the neuroglial group, serve as the resident macrophages of the nervous system, acting as primary mediators of the innate immune response [14].

**Figure 1:**
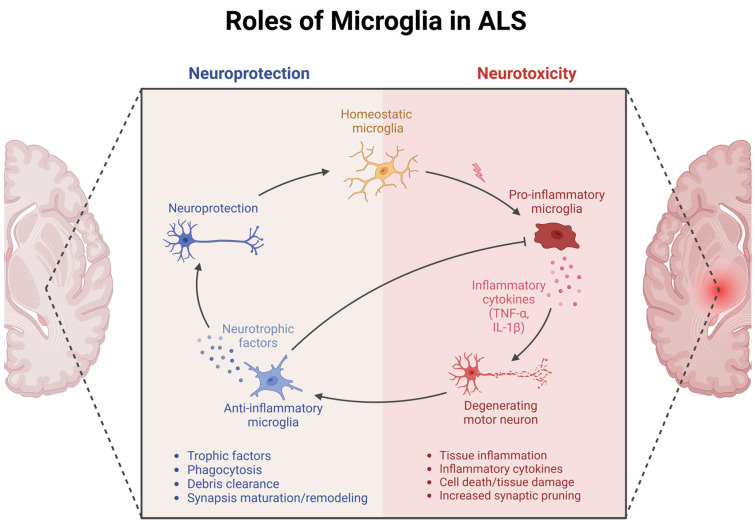
Roles of DAM in ALS: neuroprotective and neurotoxic. Under homeostatic conditions (yellow, center), microglia support neuronal health by clearing protein aggregates, releasing neurotrophic and anti-inflammatory factors, and providing immune surveillance. In ALS, however, these cells increasingly assume DAM phenotypes that can be both protective and harmful (right panel). On one hand, DAM help remove toxic aggregates and maintain synaptic integrity; on the other, they can release superoxide radicals and pro-inflammatory cytokines (e.g., TNF-α, IL-1β) and secrete factors that convert astrocytes to a neurotoxic reactive state. This triggers excessive synaptic pruning, inflames tissue, and accelerates motor neuron loss. Thus, the dynamic balance between DAM-mediated neuroprotection and neurotoxicity is pivotal in determining ALS progression. This Figure was created with biorender.com.

Deriving from yolk sac progenitors during early embryonic development, microglia migrate to the central nervous system (CNS) before the blood–brain barrier’s formation, where they proliferate and differentiate into mature cells. Under physiological conditions, microglia exist in a so-called “resting state”, characterized by small cell bodies and multiple processes, which allows them to continuously survey the surrounding environment for potential signals of infection, damage, or other homeostatic changes [14,15]. As potentially harmful or abnormal conditions are detected, microglia transition into to their “activated state”, the features of which depend on the specific stimuli inducing the response (Figure 1). Via their reversion to an ameboid morphology, they are able to travel throughout the nervous system and reach the designated site [16]. By virtue of their versatility, microglial cells cover a plethora of functions besides the surveillance role typical of the “resting state”, including innate immune defense response by engulfing debris and pathogens and by regulating inflammation [17], as well as synaptic pruning [18,19], neurogenesis support, and response to injury, where they serve as key supporters. However, microglial cells can be hijacked or destabilized under certain conditions. Particularly, malfunctioning microglia have been described as secreting chronically high levels of pro-inflammatory cytokines such as TNF-α, IL-1β, and IL-6 [20], thereby potentially contributing to the development of both psychiatric and neurodegenerative disorders including depression, Alzheimer’s, Parkinson’s, and ALS [21]. Furthermore, they may release persistently high levels of ROSs and NO or fail to clear out debris and pathogens, further promoting neuronal damage and disease progression [19,21].

One notable discovery in the context of neurodegeneration is the disease-associated microglia (DAM) phenotype, first characterized in Alzheimer’s disease (AD) [22,23] using methods like MARS-seq, smFISH, and iChiP. DAM emerge through a two-step activation process: an initial TREM2-independent phase involving the downregulation of homeostatic genes such as Cx3cr1, P2ry12, and P2ry13 and the upregulation of intermediate-state markers including B2m, ApoE, and Tyrobp, followed by a TREM2-dependent phase marked by the elevated expression of phagocytic and lipid metabolism genes like Lpl, Cst7, and CD9 [22,23]. DAM signatures, including markers such as Clec7a, CD9, Itgax, and CD63, have been identified in ALS and other neurodegenerative diseases, where they are associated with pathways linked to phagocytosis, lysosomal function, and lipid metabolism [23,24,25]. The transition to the DAM state is predominantly protective, as evidenced by findings that TREM2, a microglia-specific receptor, is critical in this shift. In ALS models, TREM2 deficiency exacerbates pathological TDP-43 inclusions, motor dysfunction, and neurodegeneration [26,27]. Similarly, the knockout of *TREM2* in chronic demyelination models impairs myelin clearance and leads to lipid accumulation. Moreover, SYK, a downstream effector of TREM2 signaling, is essential in DAM activation, with its deletion worsening neuropathology in AD and multiple sclerosis models [28]. Collectively, these findings underscore the critical role of DAM in neurodegenerative diseases, including ALS, where they appear to mediate neuroprotection by facilitating the clearance of pathological aggregates and debris, making DAM a promising target for therapeutic strategies.

## 2. The Central Role of DAM Phenotypes

In recent decades, outdated binary frameworks for microglia classification have been found to no longer reflect the complexity of microglial states, as revealed by recent advances in molecular and transcriptomic technologies [22]. These tools have uncovered a more nuanced, broad, and dynamic spectrum of microglial activation, influenced by context, environment, and disease progression [22].

**Figure 2:**
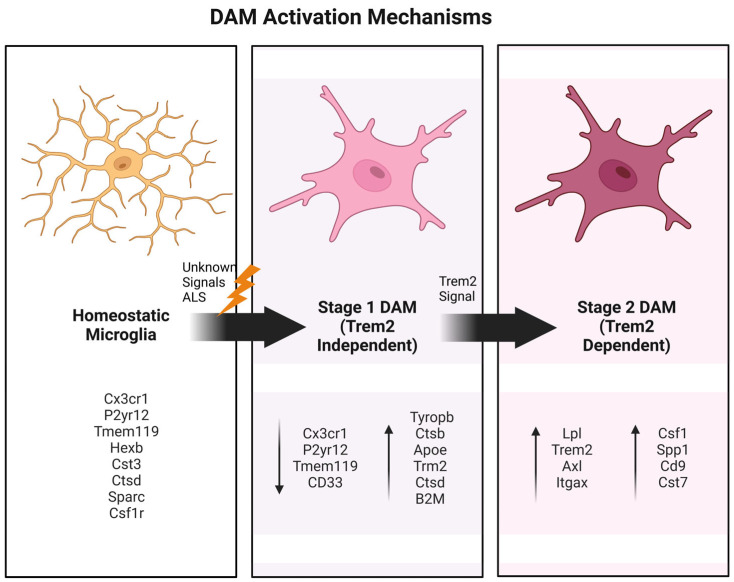
DAM activation mechanisms. In the DAM model, microglia transition from homeostatic to stage 1 (Trem2-independent) and stage 2 (Trem2-dependent), each one with their own molecular and transcriptional signature. Arrows indicate genes that are up- or downregulated at each stage [25].

In neurodegenerative diseases, such as AD [23], these observations have revealed the existence of specific activation patterns in different disease stages and regions. Subsequently described in other pathologies such as ALS, these findings have driven the field to adopt the concept of DAM to provide a more accurate and detailed characterization of microglial states [22,23,24]. DAM phenotypes are defined by unique molecular markers and functional profiles that directly reflect the demands of disease-specific environments (Figure 2).

In ALS, the emergence of DAM-like phenotypes is becoming increasingly evident. Studies of spinal cord tissue from ALS patients have identified the upregulation of genes such as TREM2, TYROBP, APOE, CD33, and MS4A, while markers like TMEM119 remain unchanged [24]. These findings point to the existence of ALS-specific DAM phenotypes that play critical roles in disease progression. This recognition challenges the reliance on outdated classifications and highlights the need for a more refined understanding of microglial dynamics in ALS [22,24,25,26,27].

The shift toward DAM phenotypes represents a transformative development in neurodegeneration research. Unlike previous oversimplified models, the DAM framework captures the diversity and specificity of microglial activation across diseases [22,23]. In ALS, this approach provides insights into the unique molecular and functional characteristics of microglia at different stages of the disease, enabling researchers to identify new therapeutic targets.

Prioritizing the study of DAM phenotypes offers the potential to redefine therapeutic strategies. By focusing on the disease-specific roles of microglia, researchers can design targeted interventions that modulate microglial activity in a precise and effective manner. This transition marks a significant advancement in ALS research, providing a more comprehensive understanding of disease mechanisms and paving the way for innovative treatments [26]. Embracing DAM phenotypes as the central framework for studying microglia is not only necessary but also transformative, promising to reshape the field and improve patient outcomes. Multiple approaches are currently under exploration.

## 3. Microglia Alterations in Human Postmortem Tissue

Research on postmortem human ALS tissue has established a link between microglia activation and ALS, though there remains some debate regarding whether microglial activation, characterized by a morphological shift from ramified or stellate forms to amoeboid shapes, increased proliferation, or the upregulation of inflammatory pathways, is consistently observed in ALS tissue. Some studies indicate that microglial proliferation is present only in select ALS cases [28,29], while others report activation in both the motor cortex [30] and spinal cord [31,32] of sporadic ALS patients. This controversy may mainly arise due to heterogeneity among ALS genetic subtypes or potential artifacts related to immunolabelling technique variability in tissue fixation and processing or tissue degradation. Additionally, normal brain aging causes substantial gene expression changes, which may influence/bias the conclusions drawn based on patients’ ages in these studies.

However, in-depth examinations of microglia in postmortem tissue are inherently limited as they provide only a snapshot of pathology at the disease’s terminal stage and may be affected by factors such as variable agonal respiration states and postmortem delays. Some of these limitations have been partially addressed through monitoring neuroinflammation in living patients. Recent innovations in PET imaging have shed light on the longitudinal dynamics of neuroinflammation in ALS patients, revealing increased inflammation in the primary motor, supplementary motor, and temporal cortices using various ligands that bind to the immune marker translocator protein 18 (TSPO) [33,34,35]. However, because TSPO also labels astrocytes and other immune cells, it is challenging to isolate microglia-specific effects. Other histology markers, such as morphological alterations and the increased expression of gliosis markers such as IBA1 and CD68, suggested a consistent hyperactivation of microglia observed in postmortem brain tissue from C9ORF72 ALS patients [32,36]. Furthermore, recent findings indicated a DAM-mediated inflammatory response in the postmortem spinal cord of TDP-43 ALS patients, as evidenced by elevated TREM2, MS4A, CD33, APOE, and TYROBP expression [22]. Similarly, sALS patients exhibited increased TMEM119-positive microglia in the motor cortex and white matter, highlighting microglial activation as a key feature in ALS [37].

Recent single-cell studies have transformed our understanding of human microglial heterogeneity, revealing diverse microglial subtypes and their roles in health and disease. A comprehensive analysis of 215,658 postmortem human microglia across various CNS regions and conditions identified distinct microglial subpopulations, suggesting divergent differentiation pathways with unique functional and metabolic shifts [38]. Certain subtypes were enriched for genes linked to neuropsychiatric disease susceptibility, while others, such as the CXCR4-enriched cluster, were associated with neuroinflammatory conditions rather than neurodegenerative diseases like ALS or FTD. These findings highlight the complexity of microglial diversity and suggest that microglia may play a limited primary role in the pathogenesis of ALS and other diseases, potentially due to less extensive GWAS annotations or disease-specific mechanisms. Other transcriptomic studies also show that microglia exhibit significant transcriptional changes in ALS, particularly in the primary motor cortex, where differentially expressed genes linked to energy metabolism and oxidative phosphorylation have been identified. Upregulated genes such as SOD1, CALM1, and COX6C, alongside downregulated genes like HSPA1A, highlight potential biomarkers associated with ALS pathogenesis. Reduced microglial interactions with astrocytes and other cells under high oxidative phosphorylation states suggest a critical role for disrupted energy metabolism in disease progression [39].

Current research in humans highlights that while microglia are implicated in ALS pathology, there remains substantial uncertainty due to methodological limitations and patient heterogeneity. Although postmortem analyses provide valuable insights, they are constrained by their static nature and potential artifacts. In vivo imaging using PET offers some advantages in studying disease progression in humans but lacks the cellular specificity needed to isolate microglial contributions. Therefore, mouse models of ALS are crucial model systems for dissecting the contribution of microglia in ALS pathogenesis.

## 4. Insights from Mouse Models

Early studies suggested that ALS developed in a non-cell-autonomous fashion [40,41,42,43,44]. Indeed, the implementation of chimeric mice showed that healthy motor neurons develop ALS-like symptoms when surrounded by mutant SOD1 microglia (mSOD1), reinforcing the concept that alteration of microglial features and activation states contributes to motor neuron injury and degeneration. The initial identification of the neurotoxic nature of mSOD1 microglia in vitro was subsequently updated by the finding, through in vivo studies, of additional unexpected neuroprotective features of ALS microglia, especially in the early stages of the disease [41,43,45].

Among animal models for ALS, those based on mutations in the SOD1 gene are particularly informative, as SOD1 mutations account for approximately 20% of fALS cases. An overview of the main mouse models used to study microglia in ALS is provided in Table 1. Key SOD1 models include the slow-progressing SOD1^G37R^ [42] and the fast-progressing SOD1^G93A^ models [46], which have been extensively used to study ALS pathology. The SOD1^G93A^ transgenic model, expressing a mutant form of Cu^2+^/Zn^2+^ human SOD1, mirrors several pathological features of ALS, including progressive motor neuron degeneration, muscle atrophy, and a reduced lifespan [46,47]. Notably, studies using this model report early microglial activation well before clinical symptoms appear, suggesting that microglia may initially respond to subtle motor neuron dysfunction signals—such as protein misfolding, oxidative stress, or mitochondrial abnormalities—rather than triggering the disease [41,48,49].

These early changes indicate that microglia might have a protective role at initial disease stages by clearing damaged cells and maintaining CNS homeostasis [49,56]. However, as ALS progresses, reactive microglia increasingly adopt a pro-inflammatory and neurotoxic phenotype, exacerbating motor neuron loss [57]. This shifting balance between neuroprotection and neurotoxicity suggests that microglial contributions in ALS are linked to disease progression rather than initiation [49]. In SOD1^G93A^ mice, DAM have been identified in the spinal cord and brainstem, emerging after motor neuron loss and displaying enhanced phagocytic activity. These microglia exhibited distinct transcriptional changes, including the upregulation of genes such as Cst7 and Itgax, and their activation appears to be triggered by danger signals from degenerating motor neurons [58].

Transcriptomic and proteomic analyses of microglia from SOD1^G93A^ mice further reveal the marked upregulation of genes related to inflammation, phagocytosis, lipid metabolism, and cell survival. For instance, pro-inflammatory cytokine genes such as TNF-α and IL-1β are highly expressed, underscoring the active role of microglia in promoting neuroinflammation within mSOD1 ALS models [59,60]. The chronic activation of microglia in the SOD1^G93A^ mouse model leads to a progressive loss of immune identity and functional efficiency. This is demonstrated by distinct proteomic signatures, a diminished phagocytic capacity, and reduced responses to immune challenges [61]. Notably, advanced disease stages are characterized by a shift toward RNA metabolism rather than immune-related functions.

Additionally, microglia in these models display impaired phagocytic capacity, potentially leading to the accumulation of apoptotic cells and toxic protein aggregates that could further harm neurons.

Experiments using selective microglial depletion or modulation of microglial states in SOD1^G93A^ mice have demonstrated delays in disease onset and lifespan extension, reinforcing the view that microglia—while initially protective—become deleterious as ALS advances [41,62,63]. Successfully characterizing the nuances of microglial activation in ALS is therefore essential in understanding disease progression and may hold therapeutic potential in modulating microglial activity to alleviate ALS symptoms.

### 4.1. Morphological Changes in Microglia in Mice

Microglia exhibit distinct morphological adaptations during the progression of ALS. In the surveillance state (S), microglia display a highly ramified morphology, which is markedly different from the less ramified forms of reactive microglia subtypes R1 and R2. In SOD1^G93A^ transgenic mice, R1 microglia comprise approximately 85% of the microglial population in the spinal cord during the early stages of ALS symptoms, whereas R2 microglia become predominant at later stages of the disease [59]. These observations suggest that the transition from S to R1 and R2 morphologies is associated with distinct phases of ALS pathology. However, as morphology is not always a reliable indicator of function, it is imperative that we validate these morphological differences through detailed functional analyses, particularly given the minimal visual distinctions between R1 and R2 microglia.

### 4.2. CX3CR1 Signaling and Microglia–Neuron Crosstalk

It is now well established that communication between neurons and microglia is essential in maintaining CNS homeostasis and mounting responses to injury. A key pathway mediating this interaction is the fractalkine–CX3CR1 signaling pathway, where CX3CR1—a receptor primarily found on microglia—responds to fractalkine, a chemokine produced by neurons [54,64]. This pathway plays a critical role in regulating microglial activity, both under normal physiological conditions and in neurodegenerative disease contexts [65].

In ALS, disruptions in fractalkine–CX3CR1 signaling have been shown to increase microglial activation and neurotoxicity. Studies in SOD1^G93A^ ALS mouse models indicate that CX3CR1 deficiency accelerates motor neuron loss and worsens disease progression, highlighting the pathway’s role in neuroprotection [54]. Conversely, restoring fractalkine–CX3CR1 signaling in these models reduces microglial activation and slows motor neuron degeneration [55].

Given its influence on microglial behavior, the fractalkine–CX3CR1 pathway represents a promising therapeutic target for modulating neuroinflammation in ALS [65]. Although targeting this pathway alone may not be sufficient to cure ALS, re-establishing effective neuron–microglia communication through CX3CR1 signaling could reduce neuroinflammation and help protect motor neurons, potentially mitigating disease progression.

### 4.3. NF-κB Pathway and Microglial Activation in ALS Progression

Seminal research by Swarup et al. [11] first implicated the nuclear factor kappa-light-chain-enhancer of activated B cells (NF-κB) pathway as a crucial factor in the pathogenesis of ALS, directly linking it to TDP-43 pathology. The study reported a fourfold increase in NF-κB levels within the spinal cords of ALS patients and demonstrated that the overexpression of TDP-43 induces the nuclear translocation of the NF-κB subunit p65. Notably, this translocation requires an additional inflammatory stimulus or “second hit”, such as lipopolysaccharide (LPS), tumor necrosis factor-alpha (TNF-α), or interleukin-1 beta (IL-1β). The pharmacological inhibition of NF-κB activity significantly reduced the vulnerability of neurons overexpressing TDP-43 to toxicity induced by microglia and glutamate, identifying NF-κB as a potential therapeutic target for ALS.

Building upon these findings, subsequent studies have explored the role of NF-κB in ALS linked to mutant superoxide dismutase 1 (SOD1). In SOD1^G93A^ mice, the microglial inhibition of NF-κB substantially delayed disease progression and extended survival by approximately 20 days [66]. Furthermore, the partial, myeloid-specific inhibition of NF-κB in mice delayed disease progression by 47%, while the complete inhibition of NF-κB in vitro restored MN survival, underscoring the essential role of NF-κB-dependent microglial activity in motor neuron death [63]. Intriguingly, NF-κB inhibition in astrocytes did not confer protective benefits and, in some cases, appeared detrimental [51], suggesting that microglia and astrocytes may influence ALS progression through distinct mechanisms.

ALS-associated microglia exhibit a distinctive pro-inflammatory activation profile. In the SOD1^G93A^ IKKβ-f/wt CSF-1R-cre+ mouse model, NF-κB inhibition led to a significant reduction in inflammatory markers—including CD68, CD86, and inducible nitric oxide synthase (iNOS)—compared to Cre-negative controls [44,49,67]. The 20-day extension in the median life span of SOD1^G93A^ mice upon NF-κB inhibition emphasizes the central role of pro-inflammatory microglia in motor neuron death in ALS [62]. Additionally, the constitutive activation of NF-κB in wild-type microglia decreased motor neuron survival by 50%, independent of SOD1 mutations, and promoted muscle atrophy, further highlighting the pivotal role of NF-κB in ALS pathogenesis [66]. Moreover, in studies utilizing CSF1R-Cre mice, researchers observed astrogliosis in addition to reduced microglial activation compared to Cre-negative control littermates, further suggesting the role of NF-κB in promoting disease progression. Interestingly, the inhibition of NF-κB in astrocytes did not benefit ALS mice; subsequent studies reported that this inhibition could, in fact, have a detrimental effect [51].

The upregulation of NF-κB activation has also been observed in tissue cultures derived from sporadic ALS (sALS) patients [7]. Other ALS-linked proteins, such as TDP-43 and fused in sarcoma (FUS), are known to interact with the NF-κB pathway, and the inhibition of NF-κB in transgenic models overexpressing these genes ameliorated ALS-related pathological features [11,68]. These findings position NF-κB as a promising therapeutic target in both sporadic and familial forms of ALS. However, the multifunctional nature of NF-κB across different cell types poses challenges for therapeutic development. This underscores the need for more detailed mechanistic studies to elucidate the pathways by which NF-κB activation leads to motor neuron degeneration [66].

### 4.4. The C9ORF72 Mouse Model

The hexanucleotide G_4_C_2_ repeat expansion (HRE) within the first intronic region of the *C9ORF72* gene represents the most common genetic cause of familial fALS in humans [3]. This expansion, which extends from physiological levels of approximately 20 repeats to pathological levels of hundreds or thousands, leads to the production of toxic dipeptide repeat proteins and RNA foci, contributing to neurodegeneration. Mouse models expressing pathological G_4_C_2_ expansions (C9-500) in a full-length human *C9ORF72* gene have been established to elucidate ALS pathogenesis mechanisms, particularly in relation to microglial function [7,50,66,67,68]. In neurons, the *C9ORF72* HRE promotes the accumulation of RNA foci and toxic protein aggregates, which accelerates motor neuron degeneration. Interestingly, such pathological features are less evident in glial cells, even though microglia exhibit the highest levels of C9ORF72 expression in the CNS [68,69].

Studies in C9orf72-deficient mice have demonstrated that microglia show abnormal activation, marked by increased pro-inflammatory cytokine production and reduced phagocytic capacity [54]. While the molecular details of these mechanisms remain largely unknown, it has been proposed that they result from an aberrant trafficking of CD80 to the cell membrane with consequent antigen-independent T-cell activation and a concomitant disruption in IL-17A-dependent inflammation [70].

Additionally, the loss of C9ORF72 directly altered the expression of activated response microglia (ARM) and interferon response microglia (IRM), which added to the notion that C9ORF72 is required for correct microglial function. Moreover, such alteration resulted in a decrease in synaptic pruning in both co-cultures and in vivo mouse models [71]. Mutations in *C9ORF72* have also been linked to impaired autophagy, a critical process for the microglial-mediated clearance of cellular debris and CNS homeostasis [52,72]. Collectively, these observations imply that microglial phenotypic changes play a significant role in the progression of C9ORF72-related ALS.

Furthermore, novel insights additionally revealed that many of the genes that are shown to be functionally impaired in *C9orf72^−/−^* mice are involved in late endosomal trafficking and lysosome function, including *Tbk1, Tmem106b, Pgrn,* and *Optn*, which are expressed in both neurons and microglia. This notion further reinforces the existence of a dual-effect mechanism in the development of ALS and raises important considerations related to the consequences of C9ORF72 loss in therapeutic contexts [68].

### 4.5. TREM2 Signaling in ALS

Triggering receptor expressed on myeloid cells 2 (TREM2) is a receptor expressed predominantly on microglial cells that plays a crucial role in regulating microglial function, particularly in response to injury and neurodegeneration [73,74]. Specifically, TREM2 signaling is involved in processes such as phagocytosis, lipid metabolism, and inflammatory responses. The dysregulation of TREM2 has been implicated in several neurodegenerative diseases, including ALS [74,75]. Specifically, TREM2 has been linked to disease-associated microglia (DAM), where it is essential in detecting and responding to pathological cues. DAM, conserved in both mice and humans, are characterized by a specific gene expression pattern, as described above, which offers a promising avenue for microglia-mediated ALS treatment [40].

In mouse models of ALS, particularly SOD1^G93A^ mice, TREM2 expression is upregulated in microglia during disease progression [60]. Studies have shown that TREM2-deficient mice exhibit increased motor neuron degeneration, heightened neuroinflammation, and enhanced microglial activation, suggesting that TREM2 may play a protective role in ALS [76]. Strikingly, the overexpression of TREM2 has been demonstrated to mitigate microglia-mediated inflammation and enhance the clearance of misfolded proteins, reinforcing the hypothesis of its neuroprotective role and indicating potential therapeutic applications in ALS [76]. Specifically, the protective effects of TREM2 are thought to be mediated by its ability to promote a shift in microglial phenotype toward a neuroprotective, anti-inflammatory state [76,77]. Therefore, targeting TREM2 signaling may represent a promising therapeutic strategy for modulating microglial activity in ALS, warranting further investigation in future studies and clinical trials.

### 4.6. Role of TREM2 and CD14 in TDP-43-Driven ALS

Recent studies have established a critical link between TDP-43 and the microglial receptors TREM2 and CD14 in the pathological progression of TDP-43-driven ALS [72,78]. TREM2 regulates microglial responses—particularly the phagocytic clearance of pathological TDP-43—through confirmed interactions in silico, in vitro, and in vivo. TREM2 deficiency diminishes microglial activation in response to TDP-43 accumulation, mirroring findings observed with amyloid-beta (Aβ) [79]. Notably, the overexpression of TDP-43 enhances neuron–microglia interactions, suggesting a “find-me” signaling mechanism mediated by TREM2. Impaired or downregulated TREM2 exacerbates ALS pathology due to reduced microglial phagocytic activity. Additionally, increasing soluble TDP-43 levels improves microglial phagocytosis, highlighting TREM2’s therapeutic potential in TDP-43-mediated neurodegeneration [79].

Parallel research identifies CD14 as another receptor through which TDP-43 activates microglia, initiating a pro-inflammatory cascade via the NF-κB pathway [80]. The selective blockade of microglial CD14 activity suppresses pro-inflammatory cytokine production in response to pathological TDP-43, reinforcing its connection to ALS pathogenesis, similarly to TREM2. While TDP-43 loss of function is associated with the accumulation of double-stranded RNAs (dsRNAs) in mouse spinal cords and in the brains of patients with C9ORF72 HRE, a direct causal link to disease progression remains elusive.

Moreover, an emerging body of evidence highlights the critical role of activated microglia in modulating perineuronal nets (PNNs) in ALS, particularly in TDP-43-driven models. Studies report that increased microgliosis and matrix metalloproteinase-9 (MMP-9) secretion correlates with the loss of PNNs, extracellular matrix structures surrounding motor neurons, which may exacerbate alpha-motor neuron degeneration and ALS symptoms in the TDP-43^Q331K^ mouse model [49]. This highlights the significant contribution of microglial activity and extracellular matrix disruption in TDP-43-mediated neurodegeneration. Insights from other ALS models, such as SOD1^G93A^, also point to PNN disruptions linked to microglial activity, suggesting a conserved mechanism [46]. Comparatively, findings in Alzheimer’s disease (AD) provide additional context, where microglia actively associate with and engulf damaged PNNs [80], with the pharmacological depletion of microglia preventing PNN loss in 5xFAD models [81]. Furthermore, it has been shown that TDP-43 mutations in microglia significantly influence miRNA release on a sex-dependent basis, with female-derived homozygous mutant microglia displaying altered miRNA profiles, including the downregulated release of miR-16-5p and miR-99a-5p, which are implicated in ALS pathogenesis. These findings suggest that microglia-derived miRNAs may act as regulators of gene expression in other cell types, opening up new avenues for understanding the role of microglia in neurodegeneration [81].

Interestingly, in the TDP-43 mouse model, rod-shaped microglia, a subtype of DAM, emerged in the motor cortex in response to neuronal hyperactivity, aligning with pyramidal dendrites and pruning excitatory synapses. These microglia, marked by genes such as TREM2 and galectin-3, played a neuroprotective role, as their absence exacerbated hyperactivity and motor dysfunction and reduced survival [82].

These observations collectively emphasize the central role of microglial receptors and extracellular matrix components in neurodegeneration, with TDP-43-driven ALS serving as a key model to explore these interactions.

## 5. Role of Microglia in Human iPSC in Vitro Systems

Rodent models have been instrumental in elucidating the roles of microglia in ALS pathogenesis; however, they come with notable limitations, primarily due to cross-species differences in microglial biology, which can reduce the translational relevance of these findings [83,84]. Moreover, while most ALS microglial studies rely on genetic rodent models, most ALS cases occur sporadically, highlighting a gap in model relevance. To bridge this gap, human-induced pluripotent stem cell (iPSC)-derived microglia from ALS patients offer a valuable complementary approach to studying microglial pathology in a more human-relevant system [85].

Advances in protocols for generating microglia from human iPSCs [86,87] have enabled researchers to explore the role of human microglia in neurodegenerative contexts with new depth. Although the application of iPSC-derived microglia in ALS research is still emerging, early investigations have focused on *C9ORF72* mutation effects in these cells [88,89,90]. Findings indicate that *C9ORF72* mutant microglia alone do not significantly harm healthy motor neurons [89]. However, when primed with lipopolysaccharide (LPS), these mutant microglia exhibit a heightened pro-inflammatory response and reduce motor neuron survival, while unprimed mutant microglia remain largely non-toxic [88,89]. Importantly, no significant alteration was found in microglia-enriched genes when *C9ORF72* microglia was grown in monocultures in the absence of surrounding CNS cell types. This neurotoxic effect is partially mediated by the increased release of MMP9 from LPS-primed *C9ORF72* mutant microglia [90].

Impaired autophagy has emerged as a hallmark of *C9ORF72* mutant and knockout iPSC-derived microglia, a dysfunction that aligns with the increased vulnerability of *C9ORF72* mutant iPSC-derived motor neurons to excitotoxic damage [90]. Similarly, recent studies have demonstrated deficient autophagy in *C9ORF72* mutant microglia, contributing to decreased motor neuron survival in co-culture through the hyperactivation of the NF-kB and NLRP3 axis. Together, these findings support the hypothesis of a dual mechanism involving both gain-of-function and haploinsufficiency effects of the hexanucleotide repeat expansion in microglia [90].

Homozygous *FUS* P525L mutations in human iPSC-derived microglia have been shown to disrupt transcriptome profiles and alter chemoreceptor signaling, with the upregulation of genes such as *P2RY6* and *GPR183.* These findings are particularly significant as mutations in the FUS gene are a known cause of ALS, contributing to its pathogenesis through both cell-autonomous and non-cell-autonomous mechanisms. The upregulated chemoreceptors, which are activated by signals from cellular damage and cholesterol metabolites, may initially facilitate protective processes such as phagocytosis and remyelination. However, their prolonged activation could lead to chronic neuroinflammation and exacerbate disease progression [91].

Other genetic causes of ALS, such as mutations in *VCP* and *PFN1*, have also been studied using iPSC-derived microglia. iPSC-derived *VCP* mutant microglia exhibit immune and lysosomal dysfunction, undergo reactive transformation, and elicit non-cell-autonomous effects on motor neurons and astrocytes through JAK-STAT signaling, partially mirroring microglial phenotypes observed in other ALS models and postmortem tissues [92]. On the other hand, iPSC-derived microglia-like cells with ALS-linked *PFN1* mutations exhibit lipid dysmetabolism, autophagy dysregulation, and impaired phagocytosis, implicating a toxic gain of function for mutant PFN1 in autophagic and endo-lysosomal pathways, with rapamycin shown to rescue phagocytic dysfunction [93].

Adding further complexity, monocyte-derived microglia from sporadic ALS patients exhibit pathological TDP-43 inclusions, a diminished phagocytic capacity, and an amplified pro-inflammatory response to lipopolysaccharides (LPSs) compared to healthy controls [94,95]. Collectively, these findings underscore the critical role of in vitro and iPSC-derived and patient-specific models in advancing our understanding of ALS pathogenesis, offering a closer approximation of human disease mechanisms.

## 6. Therapeutic Targeting of Microglia in ALS

Given the central role of microglia in ALS pathogenesis, modulating microglial activity has emerged as a promising therapeutic strategy. Multiple approaches are currently under exploration, including anti-inflammatory treatments, gene therapy, and the enhancement of phagocytosis [13]. Since excessive inflammatory responses are a major contributor to microglia-driven motor neuron degeneration, reducing microglia-driven inflammation is a primary focus in ALS research. For instance, nonsteroidal anti-inflammatory drugs (NSAIDs), such as celecoxib in combination with ciprofloxacin [95], and selective inhibitors of pro-inflammatory cytokines (e.g., IL-1β and TNF-α inhibitors) [96] are being investigated for their potential to slow disease progression, carefully considering correct administration timing and the potential risk of excessive inflammation suppression.

However, these treatments primarily target symptomatic aspects of ALS, while underlying causes may continue to drive disease progression. Thus, while symptom management is essential, addressing the root causes of ALS will be necessary for more effective long-term therapies [13]. In this regard, gene therapy offers promising potential, enabled by advances in AAV-mediated gene delivery [97] and precise gene-editing technologies like CRISPR/Cas9 [98,99]. These technologies provide new avenues to target specific microglial pathways more deeply. For example, enhancing TREM2 or CX3CR1 signaling, or modulating microglial activation via galectins [61,100], may help shift ALS-associated microglia toward a neuroprotective phenotype while reducing their neurotoxic tendencies. For example, CD109 intervention in vitro has demonstrated the ability to partially attenuate the inflammatory response and suppress TGFβ/SMAD pathway activation in both LPS-stimulated BV2 microglia and primary SOD1^G93A^ microglia, offering further insights into strategies for modulating microglial behavior in ALS [101].

Building on these insights, the therapeutic modulation of microglial phenotypes is emerging as a compelling avenue. Interventions aimed at preventing or reversing the shift from a neuroprotective to a neurotoxic state are being developed. Compounds that inhibit pro-inflammatory pathways, such as NF-κB and JAK/STAT [102], may also support microglial homeostasis and mitigate neurotoxicity.

Another promising approach involves enhancing microglial phagocytic activity. Increasing the microglial clearance of misfolded proteins and apoptotic cells could offer neuroprotective effects. To this end, small molecules designed to boost microglial phagocytic function are currently under investigation [103].

Recent studies have also highlighted microRNAs (miRNAs), particularly miR-124 [104,105], as potent regulators of microglial activity, promoting an anti-inflammatory phenotype and offering neuroprotection through mechanisms such as the C/EBP-α pathway [106]. Non-cell-free therapies, including small extracellular vesicles (sEVs) and cell secretomes, have emerged as promising platforms for delivering miRNAs in ALS. For example, mesenchymal stem cell-derived sEVs enriched with miRNAs have demonstrated the ability to modulate microglial activation and mitigate neuroinflammation in neurodegenerative models [106]. Furthermore, secretomes from motor neurons enriched with miR-124 have been shown to influence microglial states and reduce neuroinflammatory responses, highlighting their therapeutic relevance in ALS [85]. These findings highlight the potential of miRNA-based therapies delivered via sEVs or secretomes as innovative strategies to counteract microglia-driven neuroinflammation and slow disease progression in ALS.

Although these therapeutic strategies are still in the experimental phase, they hold substantial potential to modify ALS progression by addressing the mechanisms behind microglial pathological activation. Clinical trials are underway, though their scope remains limited due to high costs and procedural complexity. Nonetheless, these approaches represent significant steps toward developing targeted ALS therapies.

## 7. Conclusions

Emerging research in mouse models and human-derived iPSC systems has highlighted the pivotal role of microglia in ALS pathogenesis, revealing their dual contributions to neuroprotection and neurodegeneration. The dysregulation of critical signaling pathways, such as TREM2 and CX3CR1, has been closely linked to ALS progression, and studies in C9ORF72 models have further illuminated the complex phenotypes of microglia in the context of this disease. The growing focus on DAM-like phenotypes in ALS research represents a significant advancement, offering a precise and disease-relevant framework for understanding microglial dynamics. These phenotypes, characterized by unique molecular markers and functional properties, provide crucial insights into the roles of microglia in ALS pathology and highlight novel therapeutic targets.

Future therapeutic strategies must incorporate these emerging insights, focusing on modulating microglial activity to promote beneficial phenotypes while mitigating neurotoxic inflammation. By targeting DAM-associated pathways and building on the evolving understanding of microglial phenotypes, it may be possible to develop treatments that slow or halt disease progression. This approach represents a promising direction for addressing the challenges of ALS, a condition that remains incurable despite decades of research.

## Figures and Tables

**Table 1:** An overview of the main mouse models used to study microglia in ALS.

Mouse Model	Genetic Modification	Key Findings
SOD1^G93A^ transgenic mouse [40,43]	Mouse expresses mutant human SOD1 with G93A mutation	- Early activation of microglia before clinical symptoms - Microglia shift from neuroprotective to neurotoxic phenotype as disease progresses - Upregulation of pro-inflammatory cytokines (TNF-α, IL-1β) - Impaired phagocytic capacity of microglia - Distinct proteomic signatures on chronically activated microglia - Depletion or resting of microglia delays disease onset and extends lifespan - Loss of perineuronal nets (PNNs)
SOD1^G37R^ mouse [42]	Mouse expresses mutant human SOD1 with G37R mutation	- Slow-progressing model of ALS - Used to study disease progression and microglial contributions over time
TDP-43^Q331K^ mouse [50]	Mouse expresses mutant TDP-43 with Q331K mutation	- Loss of perineuronal nets (PNNs) exacerbates α-motor neuron degeneration and ALS symptoms - Highlights the role of extracellular matrix in neuronal protection
SOD1^G93A^; IKKβf/wt; CSF1R-Cre+ mice [49]	Mice express SOD1^G93A^ mutation with conditional NF-κB inhibition in microglia	- Reduced expression of inflammatory markers (CD68, CD86, iNOS) - Demonstrates that pro-inflammatory microglia contribute to motor neuron death - NF-κB inhibition in microglia delays disease progression
CSF1R-Cre mice [51]	Mice express Cre recombinase under the CSF1R promoter for microglia-specific gene manipulation	- Show reduced microglial activation and astrogliosis compared to controls - NF-κB inhibition in astrocytes does not improve ALS symptoms and may be detrimental - Suggests distinct roles of microglia and astrocytes in ALS progression
C9ORF72 mouse models (e.g., C9-500) [52,53]	Mice with hexanucleotide G_4_C_2_ repeat expansions in the C9ORF72 gene	- Microglia exhibit abnormal activation with increased pro-inflammatory cytokines - Impaired phagocytic function and autophagy - Accumulation of toxic protein aggregates - Contributes to motor neuron degeneration and disease progression - Indicates both gain-of-function toxicity and loss-of-function effects from C9ORF72 mutations
CX3CR1-deficient SOD1^G93A^ mice [54,55]	Mice express SOD1^G93A^ mutation with CX3CR1 receptor deficiency in microglia	- CX3CR1 deficiency exacerbates motor neuron loss - Accelerated disease progression - Highlights the importance of neuron–microglia communication via fractalkine–CX3CR1 signaling in neuroprotection

## Data Availability

No new data were generated or analyzed in this study.

## References

[1] Grad L.I., Rouleau G.A., Ravits J., Cashman N.R. (2017). Clinical Spectrum of Amyotrophic Lateral Sclerosis (ALS). Cold Spring Harb. Perspect. Med..

[2] Mehta P., Raymond J., Nair T., Han M., Punjani R., Larson T., Berry J., Mohidul S., Horton D.K. (2024). Prevalence of ALS in All 50 States in the United States, Data from the National ALS Registry, 2011–2018. Amyotroph Lateral Scler Front. Degener.

[3] Talbott E.O., Malek A.M., Lacomis D. (2016). The epidemiology of amyotrophic lateral sclerosis. Handb. Clin. Neurol..

[4] Fang F., Ingre C., Roos P., Kamel F., Piehl F. (2015). Risk Factors for Amyotrophic Lateral Sclerosis. Clin. Epidemiol..

[5] Kaur S.J., McKeown S.R., Rashid S. (2016). Mutant SOD1 Mediated Pathogenesis of Amyotrophic Lateral Sclerosis. Gene.

[6] Zhu Q., Jiang J., Gendron T.F., McAlonis-Downes M., Jiang L., Taylor A., Diaz Garcia S., Ghosh Dastidar S., Rodriguez M.J., King P. (2020). Reduced C9ORF72 Function Exacerbates Gain of Toxicity from ALS/FTD-Causing Repeat Expansion in C9orf72. Nat. Neurosci..

[7] Källstig E., McCabe B.D., Schneider B.L. (2021). The Links between ALS and NF-ΚB. Int. J. Mol. Sci..

[8] Van Den Bosch L., Van Damme P., Bogaert E., Robberecht W. (2006). The Role of Excitotoxicity in the Pathogenesis of Amyotrophic Lateral Sclerosis. Biochimica et Biophysica Acta (BBA) Mol. Basis Dis..

[9] Odierna G.L., Vucic S., Dyer M., Dickson T., Woodhouse A., Blizzard C. (2024). How Do We Get from Hyperexcitability to Excitotoxicity in Amyotrophic Lateral Sclerosis?. Brain.

[10] Spreux-Varoquaux O., Bensimon G., Lacomblez L., Salachas F., Pradat P.F., Le Forestier N., Marouan A., Dib M., Meininger V. (2002). Glutamate Levels in Cerebrospinal Fluid in Amyotrophic Lateral Sclerosis: A Reappraisal Using a New HPLC Method with Coulometric Detection in a Large Cohort of Patients. J. Neurol. Sci..

[11] Swarup V., Phaneuf D., Dupré N., Petri S., Strong M., Kriz J., Julien J.-P. (2011). Deregulation of TDP-43 in Amyotrophic Lateral Sclerosis Triggers Nuclear Factor ΚB–Mediated Pathogenic Pathways. J. Exp. Med..

[12] Patel A., Lee H.O., Jawerth L., Maharana S., Jahnel M., Hein M.Y., Stoynov S., Mahamid J., Saha S., Franzmann T.M. (2015). A Liquid-to-Solid Phase Transition of the ALS Protein FUS Accelerated by Disease Mutation. Cell.

[13] Akçimen F., Lopez E.R., Landers J.E., Nath A., Chiò A., Chia R., Traynor B.J. (2023). Amyotrophic Lateral Sclerosis: Translating Genetic Discoveries into Therapies. Nat. Rev. Genet..

[14] Ginhoux F., Prinz M. (2015). Origin of Microglia: Current Concepts and Past Controversies. Cold Spring Harb. Perspect. Biol..

[15] Kierdorf K., Prinz M. (2013). Factors Regulating Microglia Activation. Front. Cell Neurosci..

[16] Hanisch U.-K., Kettenmann H. (2007). Microglia: Active Sensor and Versatile Effector Cells in the Normal and Pathologic Brain. Nat. Neurosci..

[17] Davalos D., Grutzendler J., Yang G., Kim J.V., Zuo Y., Jung S., Littman D.R., Dustin M.L., Gan W.-B. (2005). ATP Mediates Rapid Microglial Response to Local Brain Injury in Vivo. Nat. Neurosci..

[18] Paolicelli R.C., Bolasco G., Pagani F., Maggi L., Scianni M., Panzanelli P., Giustetto M., Ferreira T.A., Guiducci E., Dumas L. (2011). Synaptic Pruning by Microglia Is Necessary for Normal Brain Development. Science.

[19] Sierra A., Encinas J.M., Deudero J.J.P., Chancey J.H., Enikolopov G., Overstreet-Wadiche L.S., Tsirka S.E., Maletic-Savatic M. (2010). Microglia Shape Adult Hippocampal Neurogenesis through Apoptosis-Coupled Phagocytosis. Cell Stem Cell.

[20] Philips T., Robberecht W. (2011). Neuroinflammation in Amyotrophic Lateral Sclerosis: Role of Glial Activation in Motor Neuron Disease. Lancet Neurol..

[21] Frick L.R., Williams K., Pittenger C. (2013). Microglial Dysregulation in Psychiatric Disease. Clin. Dev. Immunol..

[22] Jauregui C., Blanco-Luquin I., Macías M., Roldan M., Caballero C., Pagola I., Mendioroz M., Jericó I. (2023). Exploring the Disease-Associated Microglia State in Amyotrophic Lateral Sclerosis. Biomedicines.

[23] Hou J., Chen Y., Grajales-Reyes G., Colonna M. (2022). TREM2 Dependent and Independent Functions of Microglia in Alzheimer’s Disease. Mol. Neurodegener..

[24] Takahashi K. (2023). Microglial Heterogeneity in Amyotrophic Lateral Sclerosis. J. Neuropathol. Exp. Neurol..

[25] Deczkowska A., Keren-Shaul H., Weiner A., Colonna M., Schwartz M., Amit I. (2018). Disease-Associated Microglia: A Universal Immune Sensor of Neurodegeneration. Cell.

[26] Ennerfelt H., Frost E.L., Shapiro D.A., Holliday C., Zengeler K.E., Voithofer G., Bolte A.C., Lammert C.R., Kulas J.A., Ulland T.K. (2022). SYK Coordinates Neuroprotective Microglial Responses in Neurodegenerative Disease. Cell.

[27] Keren-Shaul H., Spinrad A., Weiner A., Matcovitch-Natan O., Dvir-Szternfeld R., Ulland T.K., David E., Baruch K., Lara-Astaiso D., Toth B. (2017). A Unique Microglia Type Associated with Restricting Development of Alzheimer’s Disease. Cell.

[28] Spiller K.J., Restrepo C.R., Khan T., Dominique M.A., Fang T.C., Canter R.G., Roberts C.J., Miller K.R., Ransohoff R.M., Trojanowski J.Q. (2018). Microglia-Mediated Recovery from ALS-Relevant Motor Neuron Degeneration in a Mouse Model of TDP-43 Proteinopathy. Nat. Neurosci..

[29] Tam O.H., Rozhkov N.V., Shaw R., Kim D., Hubbard I., Fennessey S., Propp N., Fagegaltier D., Harris B.T., Ostrow L.W. (2019). Postmortem Cortex Samples Identify Distinct Molecular Subtypes of ALS: Retrotransposon Activation, Oxidative Stress, and Activated Glia. Cell Rep..

[30] Dols-Icardo O., Montal V., Sirisi S., López-Pernas G., Cervera-Carles L., Querol-Vilaseca M., Muñoz L., Belbin O., Alcolea D., Molina-Porcel L. (2020). Motor Cortex Transcriptome Reveals Microglial Key Events in Amyotrophic Lateral Sclerosis. Neurol. Neuroimmunol. Neuroinflamm..

[31] D’Erchia A.M., Gallo A., Manzari C., Raho S., Horner D.S., Chiara M., Valletti A., Aiello I., Mastropasqua F., Ciaccia L. (2017). Massive Transcriptome Sequencing of Human Spinal Cord Tissues Provides New Insights into Motor Neuron Degeneration in ALS. Sci. Rep..

[32] Brettschneider J., Toledo J.B., Van Deerlin V.M., Elman L., McCluskey L., Lee V.M.-Y., Trojanowski J.Q. (2012). Microglial Activation Correlates with Disease Progression and Upper Motor Neuron Clinical Symptoms in Amyotrophic Lateral Sclerosis. PLoS ONE.

[33] Corcia P., Tauber C., Vercoullie J., Arlicot N., Prunier C., Praline J., Nicolas G., Venel Y., Hommet C., Baulieu J.-L. (2012). Molecular Imaging of Microglial Activation in Amyotrophic Lateral Sclerosis. PLoS ONE.

[34] Alshikho M.J., Zürcher N.R., Loggia M.L., Cernasov P., Reynolds B., Pijanowski O., Chonde D.B., Izquierdo Garcia D., Mainero C., Catana C. (2018). Integrated Magnetic Resonance Imaging and [ ^11^ C]-PBR28 Positron Emission Tomographic Imaging in Amyotrophic Lateral Sclerosis. Ann. Neurol..

[35] Turner M.R., Cagnin A., Turkheimer F.E., Miller C.C.J., Shaw C.E., Brooks D.J., Leigh P.N., Banati R.B. (2004). Evidence of Widespread Cerebral Microglial Activation in Amyotrophic Lateral Sclerosis: An [11C](R)-PK11195 Positron Emission Tomography Study. Neurobiol. Dis..

[36] Cardenas A.M., Sarlls J.E., Kwan J.Y., Bageac D., Gala Z.S., Danielian L.E., Ray-Chaudhury A., Wang H.-W., Miller K.L., Foxley S. (2017). Pathology of Callosal Damage in ALS: An Ex-Vivo, 7 T Diffusion Tensor MRI Study. Neuroimage Clin..

[37] Togawa N., Ayaki T., Yoshii D., Maki T., Sawamoto N., Takahashi R. (2024). TMEM119-Positive Microglia Were Increased in the Brains of Patients with Amyotrophic Lateral Sclerosis. Neurosci. Lett..

[38] Tuddenham J.F., Taga M., Haage V., Marshe V.S., Roostaei T., White C., Lee A.J., Fujita M., Khairallah A., Zhang Y. (2024). A Cross-Disease Resource of Living Human Microglia Identifies Disease-Enriched Subsets and Tool Compounds Recapitulating Microglial States. Nat. Neurosci..

[39] Shen D., Ji Y., Qiu C., Wang K., Gao Z., Liu B., Shen Y., Gong L., Yang X., Chen X. (2024). Single-Cell RNA Sequencing Analysis of Microglia Dissected the Energy Metabolism and Revealed Potential Biomarkers in Amyotrophic Lateral Sclerosis. Mol. Neurobiol..

[40] Xiao Q., Zhao W., Beers D.R., Yen A.A., Xie W., Henkel J.S., Appel S.H. (2007). Mutant SOD1 ^G93A^ Microglia Are More Neurotoxic Relative to Wild-type Microglia. J. Neurochem..

[41] Boilleée S., Yamanaka K., Lobsiger C.S., Copeland N.G., Jenkins N.A., Kassiotis G., Kollias G., Cleveland D.W. (2006). Onset and Progression in Inherited ALS Determined by Motor Neurons and Microglia. Science.

[42] Beers D.R., Henkel J.S., Xiao Q., Zhao W., Wang J., Yen A.A., Siklos L., McKercher S.R., Appel S.H. (2006). Wild-Type Microglia Extend Survival in PU.1 Knockout Mice with Familial Amyotrophic Lateral Sclerosis. Proc. Natl. Acad. Sci..

[43] Liao B., Zhao W., Beers D.R., Henkel J.S., Appel S.H. (2012). Transformation from a Neuroprotective to a Neurotoxic Microglial Phenotype in a Mouse Model of ALS. Exp. Neurol..

[44] Beers D.R., Henkel J.S., Zhao W., Wang J., Appel S.H. (2008). CD4+ T Cells Support Glial Neuroprotection, Slow Disease Progression, and Modify Glial Morphology in an Animal Model of Inherited ALS. Proc. Natl. Acad. Sci..

[45] Tu P.H., Raju P., Robinson K.A., Gurney M.E., Trojanowski J.Q., Lee V.M. (1996). Transgenic Mice Carrying a Human Mutant Superoxide Dismutase Transgene Develop Neuronal Cytoskeletal Pathology Resembling Human Amyotrophic Lateral Sclerosis Lesions. Proc. Natl. Acad. Sci..

[46] Gurney M.E., Pu H., Chiu A.Y., Dal Canto M.C., Polchow C.Y., Alexander D.D., Caliendo J., Hentati A., Kwon Y.W., Deng H.-X. (1994). Motor Neuron Degeneration in Mice That Express a Human Cu,Zn Superoxide Dismutase Mutation. Science.

[47] Carson M.J., Crane J., Xie A.X. (2008). Modeling CNS Microglia: The Quest to Identify Predictive Models. Drug Discov. Today Dis. Models.

[48] Henkel J.S., Beers D.R., Zhao W., Appel S.H. (2009). Microglia in ALS: The Good, The Bad, and The Resting. J. Neuroimmune Pharmacol..

[49] Cheung S.W., Bhavnani E., Simmons D.G., Bellingham M.C., Noakes P.G. (2024). Perineuronal Nets Are Phagocytosed by MMP-9 Expressing Microglia and Astrocytes in the SOD1 ^G93A^ ALS Mouse Model. Neuropathol. Appl. Neurobiol..

[50] Crosio C., Valle C., Casciati A., Iaccarino C., Carrì M.T. (2011). Astroglial Inhibition of NF-ΚB Does Not Ameliorate Disease Onset and Progression in a Mouse Model for Amyotrophic Lateral Sclerosis (ALS). PLoS ONE.

[51] Nguyen L., Montrasio F., Pattamatta A., Tusi S.K., Bardhi O., Meyer K.D., Hayes L., Nakamura K., Banez-Coronel M., Coyne A. (2020). Antibody Therapy Targeting RAN Proteins Rescues C9 ALS/FTD Phenotypes in C9orf72 Mouse Model. Neuron.

[52] Nguyen L., Laboissonniere L.A., Guo S., Pilotto F., Scheidegger O., Oestmann A., Hammond J.W., Li H., Hyysalo A., Peltola R. (2020). Survival and Motor Phenotypes in FVB C9-500 ALS/FTD BAC Transgenic Mice Reproduced by Multiple Labs. Neuron.

[53] Zhang J., Liu Y., Liu X., Li S., Cheng C., Chen S., Le W. (2018). Dynamic Changes of CX3CL1/CX3CR1 Axis during Microglial Activation and Motor Neuron Loss in the Spinal Cord of ALS Mouse Model. Transl. Neurodegener..

[54] Liu C., Hong K., Chen H., Niu Y., Duan W., Liu Y., Ji Y., Deng B., Li Y., Li Z. (2019). Evidence for a Protective Role of the CX3CL1/CX3CR1 Axis in a Model of Amyotrophic Lateral Sclerosis. Biol. Chem..

[55] Ransohoff R.M., Perry V.H. (2009). Microglial Physiology: Unique Stimuli, Specialized Responses. Annu. Rev. Immunol..

[56] Murray P.J., Wynn T.A. (2011). Protective and Pathogenic Functions of Macrophage Subsets. Nat. Rev. Immunol..

[57] Chen L.-X., Zhang M.-D., Xu H.-F., Ye H.-Q., Chen D.-F., Wang P.-S., Bao Z.-W., Zou S.-M., Lv Y.-T., Wu Z.-Y. (2024). Single-Nucleus RNA Sequencing Reveals the Spatiotemporal Dynamics of Disease-Associated Microglia in Amyotrophic Lateral Sclerosis. Research.

[58] Sousa C., Golebiewska A., Poovathingal S.K., Kaoma T., Pires-Afonso Y., Martina S., Coowar D., Azuaje F., Skupin A., Balling R. (2018). Single-cell Transcriptomics Reveals Distinct Inflammation-induced Microglia Signatures. EMBO Rep..

[59] MacLean M., Juranek J., Cuddapah S., López-Díez R., Ruiz H.H., Hu J., Frye L., Li H., Gugger P.F., Schmidt A.M. (2021). Microglia RAGE Exacerbates the Progression of Neurodegeneration within the SOD1^G93A^ Murine Model of Amyotrophic Lateral Sclerosis in a Sex-Dependent Manner. J. Neuroinflamm..

[60] Barreto-Núñez R., Béland L., Boutej H., Picher-Martel V., Dupré N., Barbeito L., Kriz J. (2024). Chronically Activated Microglia in <scp>ALS</Scp> Gradually Lose Their Immune Functions and Develop Unconventional Proteome. Glia.

[61] García-Revilla J., Boza-Serrano A., Espinosa-Oliva A.M., Soto M.S., Deierborg T., Ruiz R., de Pablos R.M., Burguillos M.A., Venero J.L. (2022). Galectin-3, a Rising Star in Modulating Microglia Activation under Conditions of Neurodegeneration. Cell Death Dis..

[62] Rodríguez-Gómez J.A., Kavanagh E., Engskog-Vlachos P., Engskog M.K.R., Herrera A.J., Espinosa-Oliva A.M., Joseph B., Hajji N., Venero J.L., Burguillos M.A. (2020). Microglia: Agents of the CNS Pro-Inflammatory Response. Cells.

[63] Lee S., Varvel N.H., Konerth M.E., Xu G., Cardona A.E., Ransohoff R.M., Lamb B.T. (2010). CX3CR1 Deficiency Alters Microglial Activation and Reduces Beta-Amyloid Deposition in Two Alzheimer’s Disease Mouse Models. Am. J. Pathol..

[64] Lopez-Lopez A., Gamez J., Syriani E., Morales M., Salvado M., Rodríguez M.J., Mahy N., Vidal-Taboada J.M. (2014). CX3CR1 Is a Modifying Gene of Survival and Progression in Amyotrophic Lateral Sclerosis. PLoS ONE.

[65] Frakes A.E., Ferraiuolo L., Haidet-Phillips A.M., Schmelzer L., Braun L., Miranda C.J., Ladner K.J., Bevan A.K., Foust K.D., Godbout J.P. (2014). Microglia Induce Motor Neuron Death via the Classical NF-ΚB Pathway in Amyotrophic Lateral Sclerosis. Neuron.

[66] Beers D.R., Henkel J.S., Zhao W., Wang J., Huang A., Wen S., Liao B., Appel S.H. (2011). Endogenous Regulatory T Lymphocytes Ameliorate Amyotrophic Lateral Sclerosis in Mice and Correlate with Disease Progression in Patients with Amyotrophic Lateral Sclerosis. Brain.

[67] Uranishi H., Tetsuka T., Yamashita M., Asamitsu K., Shimizu M., Itoh M., Okamoto T. (2001). Involvement of the Pro-Oncoprotein TLS (Translocated in Liposarcoma) in Nuclear Factor-ΚB P65-Mediated Transcription as a Coactivator. J. Biol. Chem..

[68] O’Rourke J.G., Bogdanik L., Yáñez A., Lall D., Wolf A.J., Muhammad A.K.M.G., Ho R., Carmona S., Vit J.P., Zarrow J. (2016). *C9orf72* Is Required for Proper Macrophage and Microglial Function in Mice. Science.

[69] DeJesus-Hernandez M., Finch N.A., Wang X., Gendron T.F., Bieniek K.F., Heckman M.G., Vasilevich A., Murray M.E., Rousseau L., Weesner R. (2017). In-Depth Clinico-Pathological Examination of RNA Foci in a Large Cohort of C9ORF72 Expansion Carriers. Acta Neuropathol..

[70] Limone F., Couto A., Wang J.-Y., Zhang Y., McCourt B., Huang C., Minkin A., Jani M., McNeer S., Keaney J. (2024). Myeloid and Lymphoid Expression of *C9orf72* Regulates IL-17A Signaling in Mice. Sci. Transl. Med..

[71] Lall D., Lorenzini I., Mota T.A., Bell S., Mahan T.E., Ulrich J.D., Davtyan H., Rexach J.E., Muhammad A.K.M.G., Shelest O. (2021). C9orf72 Deficiency Promotes Microglial-Mediated Synaptic Loss in Aging and Amyloid Accumulation. Neuron.

[72] Liu Y., Pattamatta A., Zu T., Reid T., Bardhi O., Borchelt D.R., Yachnis A.T., Ranum L.P.W. (2016). C9orf72 BAC Mouse Model with Motor Deficits and Neurodegenerative Features of ALS/FTD. Neuron.

[73] Tagliatti E., Desiato G., Mancinelli S., Bizzotto M., Gagliani M.C., Faggiani E., Hernández-Soto R., Cugurra A., Poliseno P., Miotto M. (2024). Trem2 Expression in Microglia Is Required to Maintain Normal Neuronal Bioenergetics during Development. Immunity.

[74] Qin Q., Teng Z., Liu C., Li Q., Yin Y., Tang Y. (2021). TREM2, Microglia, and Alzheimer’s Disease. Mech. Ageing Dev..

[75] Xie M., Zhao S., Bosco D.B., Nguyen A., Wu L. (2022). Microglial TREM2 in Amyotrophic Lateral Sclerosis. Dev. Neurobiol..

[76] Liu W., Taso O., Wang R., Bayram S., Graham A.C., Garcia-Reitboeck P., Mallach A., Andrews W.D., Piers T.M., Botia J.A. (2020). *Trem2* Promotes Anti-Inflammatory Responses in Microglia and Is Suppressed under pro-Inflammatory Conditions. Hum. Mol. Genet..

[77] Prater K.E., Latimer C.S., Jayadev S. (2022). Glial TDP-43 and TDP-43 Induced Glial Pathology, Focus on Neurodegenerative Proteinopathy Syndromes. Glia.

[78] Xie M., Liu Y.U., Zhao S., Zhang L., Bosco D.B., Pang Y.-P., Zhong J., Sheth U., Martens Y.A., Zhao N. (2022). TREM2 Interacts with TDP-43 and Mediates Microglial Neuroprotection against TDP-43-Related Neurodegeneration. Nat. Neurosci..

[79] Mills W.A., Eyo U.B. (2023). TREMble Before TREM2: The Mighty Microglial Receptor Conferring Neuroprotective Properties in TDP-43 Mediated Neurodegeneration. Neurosci. Bull..

[80] Zhao W., Beers D.R., Bell S., Wang J., Wen S., Baloh R.H., Appel S.H. (2015). TDP-43 Activates Microglia through NF-ΚB and NLRP3 Inflammasome. Exp. Neurol..

[81] Christoforidou E., Moody L., Joilin G., Simoes F.A., Gordon D., Talbot K., Hafezparast M. (2024). An ALS-Associated Mutation Dysregulates Microglia-Derived Extracellular MicroRNAs in a Sex-Specific Manner. Dis. Model. Mech..

[82] Xie M., Miller A.S., Pallegar P.N., Umpierre A., Liang Y., Wang N., Zhang S., Nagaraj N.K., Fogarty Z.C., Ghayal N.B. (2024). Rod-Shaped Microglia Interact with Neuronal Dendrites to Regulate Cortical Excitability in TDP-43 Related. bioRxiv.

[83] Masuda T., Sankowski R., Staszewski O., Böttcher C., Amann L., Sagar, Scheiwe C., Nessler S., Kunz P., van Loo G. (2019). Spatial and Temporal Heterogeneity of Mouse and Human Microglia at Single-Cell Resolution. Nature.

[84] Geirsdottir L., David E., Keren-Shaul H., Weiner A., Bohlen S.C., Neuber J., Balic A., Giladi A., Sheban F., Dutertre C.-A. (2019). Cross-Species Single-Cell Analysis Reveals Divergence of the Primate Microglia Program. Cell.

[85] Abud E.M., Ramirez R.N., Martinez E.S., Healy L.M., Nguyen C.H.H., Newman S.A., Yeromin A.V., Scarfone V.M., Marsh S.E., Fimbres C. (2017). IPSC-Derived Human Microglia-like Cells to Study Neurological Diseases. Neuron.

[86] Muffat J., Li Y., Yuan B., Mitalipova M., Omer A., Corcoran S., Bakiasi G., Tsai L.-H., Aubourg P., Ransohoff R.M. (2016). Efficient Derivation of Microglia-like Cells from Human Pluripotent Stem Cells. Nat. Med..

[87] Lorenzini I., Alsop E., Levy J., Gittings L.M., Lall D., Rabichow B.E., Moore S., Pevey R., Bustos L.M., Burciu C. (2023). Moderate Intrinsic Phenotypic Alterations in C9orf72 ALS/FTD IPSC-Microglia despite the Presence of C9orf72 Pathological Features. Front. Cell Neurosci..

[88] Vahsen B.F., Nalluru S., Morgan G.R., Farrimond L., Carroll E., Xu Y., Cramb K.M.L., Amein B., Scaber J., Katsikoudi A. (2023). C9orf72-ALS Human IPSC Microglia Are pro-Inflammatory and Toxic to Co-Cultured Motor Neurons via MMP9. Nat. Commun..

[89] Banerjee P., Mehta A.R., Nirujogi R.S., Cooper J., James O.G., Nanda J., Longden J., Burr K., McDade K., Salzinger A. (2023). Cell-Autonomous Immune Dysfunction Driven by Disrupted Autophagy in *C9orf72* -ALS IPSC-Derived Microglia Contributes to Neurodegeneration. Sci. Adv..

[90] Funes S., Jung J., Gadd D.H., Mosqueda M., Zhong J., Shankaracharya, Unger M., Stallworth K., Cameron D., Rotunno M.S. (2024). Expression of ALS-PFN1 Impairs Vesicular Degradation in IPSC-Derived Microglia. Nat. Commun..

[91] Clarke B.E., Ziff O.J., Tyzack G., Petrić Howe M., Wang Y., Klein P., Smith C.A., Hall C.A., Helmy A., Howell M. (2024). Human VCP Mutant ALS/FTD Microglia Display Immune and Lysosomal Phenotypes Independently of GPNMB. Mol. Neurodegener..

[92] Kerk S.Y., Bai Y., Smith J., Lalgudi P., Hunt C., Kuno J., Nuara J., Yang T., Lanza K., Chan N. (2022). Homozygous ALS-Linked FUS P525L Mutations Cell- Autonomously Perturb Transcriptome Profile and Chemoreceptor Signaling in Human IPSC Microglia. Stem Cell Reports.

[93] Noh M.-Y., Kwon M.-S., Oh K.-W., Nahm M., Park J., Kim Y.-E., Ki C.-S., Jin H.K., Bae J., Kim S.H. (2023). Role of NCKAP1 in the Defective Phagocytic Function of Microglia-Like Cells Derived from Rapidly Progressing Sporadic ALS. Mol. Neurobiol..

[94] Quek H., Cuní-López C., Stewart R., Colletti T., Notaro A., Nguyen T.H., Sun Y., Guo C.C., Lupton M.K., Roberts T.L. (2022). ALS Monocyte-Derived Microglia-like Cells Reveal Cytoplasmic TDP-43 Accumulation, DNA Damage, and Cell-Specific Impairment of Phagocytosis Associated with Disease Progression. J. Neuroinflamm..

[95] Salomon-Zimri S., Pushett A., Russek-Blum N., Van Eijk R.P.A., Birman N., Abramovich B., Eitan E., Elgrart K., Beaulieu D., Ennist D.L. (2023). Combination of Ciprofloxacin/Celecoxib as a Novel Therapeutic Strategy for ALS. Amyotroph. Lateral Scler. Front. Degener..

[96] Lam L., Halder R.C., Montoya D.J., Rubbi L., Rinaldi A., Sagong B., Weitzman S., Rubattino R., Singh R.R., Pellegrini M. (2015). Anti-Inflammatory Therapies of Amyotrophic Lateral Sclerosis Guided by Immune Pathways. Am. J. Neurodegener. Dis..

[97] Mueller C., Berry J.D., McKenna-Yasek D.M., Gernoux G., Owegi M.A., Pothier L.M., Douthwright C.L., Gelevski D., Luppino S.D., Blackwood M. (2020). *SOD1* Suppression with Adeno-Associated Virus and MicroRNA in Familial ALS. New Engl. J. Med..

[98] Chen Y.A., Kankel M.W., Hana S., Lau S.K., Zavodszky M.I., McKissick O., Mastrangelo N., Dion J., Wang B., Ferretti D. (2023). In Vivo Genome Editing Using Novel AAV-PHP Variants Rescues Motor Function Deficits and Extends Survival in a SOD1-ALS Mouse Model. Gene Ther..

[99] McCallister T.X., Lim C.K.W., Singh M., Zhang S., Ahsan N.S., Terpstra W.M., Xiong A.Y., Zeballos Castro M.A., Powell J.E., Drnevich J. (2025). A High-Fidelity CRISPR-Cas13 System Improves Abnormalities Associated with C9ORF72-Linked ALS/FTD. Nat. Commun..

[100] Baird M.C., Likhite S.B., Vetter T.A., Caporale J.R., Girard H.B., Roussel F.S., Howard A.E., Schwartz M.K., Reed A.R., Kaleem A. (2024). Combination AAV Therapy with Galectin-1 and SOD1 Downregulation Demonstrates Superior Therapeutic Effect in a Severe ALS Mouse Model. Mol. Ther. Methods Clin. Dev..

[101] Li Z., Zhang Y., Li D., Du X., Chen L., Guo Y. (2025). Microglial Upregulation of CD109 Expression in Spinal Cord of Amyotrophic Lateral Sclerosis Mouse Model and Its Role in Modulating Inflammation and TGFβ/SMAD Pathway. Neuroscience.

[102] Guo Q., Jin Y., Chen X., Ye X., Shen X., Lin M., Zeng C., Zhou T., Zhang J. (2024). NF-ΚB in Biology and Targeted Therapy: New Insights and Translational Implications. Signal Transduct. Target. Ther..

[103] Gao C., Jiang J., Tan Y., Chen S. (2023). Microglia in Neurodegenerative Diseases: Mechanism and Potential Therapeutic Targets. Signal Transduct. Target. Ther..

[104] Zhao J., He Z., Wang J. (2021). MicroRNA-124: A Key Player in Microglia-Mediated Inflammation in Neurological Diseases. Front. Cell Neurosci..

[105] Ponomarev E.D., Veremeyko T., Barteneva N., Krichevsky A.M., Weiner H.L. (2011). MicroRNA-124 Promotes Microglia Quiescence and Suppresses EAE by Deactivating Macrophages via the C/EBP-α–PU.1 Pathway. Nat. Med..

[106] Li S., Liu J., Liu S., Jiao W., Wang X. (2021). Mesenchymal Stem Cell-Derived Extracellular Vesicles Prevent the Development of Osteoarthritis via the CircHIPK3/MiR-124-3p/MYH9 Axis. J. Nanobiotechnol..

